# Dr. Willem Kolff: The Father of the Artificial Kidney

**DOI:** 10.7759/cureus.69098

**Published:** 2024-09-10

**Authors:** Neeraj Sharma, Eddie Khav, Aisha Elahmadi, John Ong, Sahil Parag

**Affiliations:** 1 Department of Medicine, Division of Nephrology, University of Southern California Keck School of Medicine, Los Angeles, USA

**Keywords:** artificial kidney, general nephrology dialysis and transplanation, historical vignette, kеywords: dialysis, nephrology

## Abstract

Dr. Willem J. Kolff (February 14, 1911-February 11, 2009) is widely considered the father of dialysis. In addition, his innovations also included the artificial heart and lung, which earned him the title “the Father of Artificial Organs”. In due course, his artificial kidney evolved into modern dialysis, a procedure that filters and purifies blood using an extracorporeal circuit, now a life-sustaining treatment for patients with end-stage kidney failure. Furthermore, his membrane oxygenator, which provided a method to add oxygen to blood as it passed through a machine, is still used in heart-lung machines during open-heart surgery. He is also known for his work in developing the artificial heart (although it now carries the name of his student, Dr. Robert Jarvik), which was used in subsequent designs, as a bridge to heart transplantation. Thanks to his work on the artificial kidney, millions of patients worldwide have benefited from life-sustaining hemodialysis. It can also be argued that Dr. Kolff’s introduction of dialysis in 1943 marks the dawn of modern nephrology.

## Introduction and background

The history of dialysis dates back to Thomas Graham (1805-1869), a chemistry professor at the University College London. Between 1846 and 1861, he published a series of papers on the nature of the diffusion of gases and osmotic fluids [2]. His concept of the semipermeable membrane and the diffusion of substances eventually became the foundation of dialysis. The first description of dialysis was published in 1913 by Dr. John Abel and colleagues from Johns Hopkins University, titled “On the Removal of Diffusible Substances From the Circulating Blood by Means of Dialysis, in the Transactions of the Association of American Physicians. Abel and his colleagues were the first scientists to apply the principle of dialysis for the removal of substances from the blood. His 1915 dialyzer, called a “vivi-diffusion apparatus,” contained 16 tubes of collodion membrane in a glass cylinder through which anticoagulated blood (using hiridin) passed through for dialysis.

Used mostly in dogs, this newly constructed device was named the “artificial kidney”. However, Abel and his colleagues were never able to apply this technology to humans. The credit for performing the first human dialysis belongs to Dr. Georg Hass (1886-1971). In 1924, Georg Hass performed the first human kidney hemodialysis by using a modified version of the Abel-type dialyzer. This initial procedure lasted only 15 minutes since the idea was only to demonstrate its safety and reliability. With the Haas dialyzer, blood passed through collodion tubes arranged in eight parallel cylinders in contact with the exchanged fluid. In 1928, Haas reported the results of three blood cleansings (Blutwaschungen) in two patients with chronic kidney failure. Unfortunately, due to a lack of support, Dr. Haas was forced to discontinue his work in the field of dialysis [2]. The next breakthrough happened in 1937 when Dr. William Thalhimer (1884-1961) discovered that cellulose-hydrate, a man-made material used in the sausage industry, also called cellophane, would be useful as a diffusible membrane.

**Figure 1:**
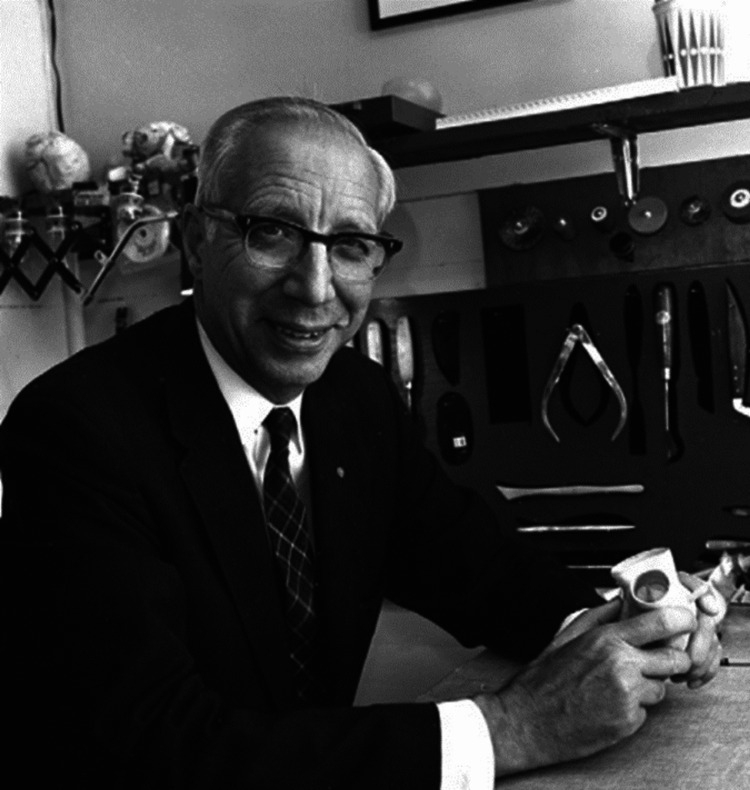
Dr. WIllem Kolff in the late 1970s Source: [1]

Fifteen years after the last human dialysis performed by Georg Haas in Giessen, Germany, Dr. Willem Kolff (Figure 1), in 1943, made the breakthrough that had stubbornly eluded Georg Haas. Dr. Kolff’s dialysis machine used cellophane tubing wrapped around a rotating drum immersed in a dialyzing solution [3]. Dr. Kolff’s version of the artificial kidney, and its principles of operation and large surface area, received worldwide acceptance in the 1950s. Over time, his artificial kidney evolved into the modern dialysis machine, which has improved the quality of life and life expectancy of millions of people with end-stage kidney disease. Figure 1 shows a photograph of Dr. Kolff in the late 1970s.

## Review

Early life

Born in Leiden, Netherlands on February 14, 1911, Dr. Kolff decided at an early age to follow in his father’s footsteps, a physician who directed the tuberculosis sanatorium [4]. As a teenager, Willem was influenced by his father’s medical practice, especially his difficulties in dealing with patients dying of tuberculosis. Kolff decided to pursue medicine and started his medical studies at the University of Leiden in 1930. After receiving his degree in 1938, he began his postgraduate studies under Dr. Leonard Polak Daniels at the University of Groningen. Dr. Daniels had a profound influence on Kolff’s approach to patients. In October 1938, Kolff witnessed the death of a 22-year-old man, Jan Bruning, from kidney failure. As a result, Kolff became determined to search for methods to treat kidney failure. As concisely recounted in his 1946 text “New Ways of Treating Anemia”, Kolff stated: “The patient could have been saved if only 20 g of urea could have been removed from his blood every day.” Furthermore, he knew that the uremic syndrome was an intoxication induced by retained metabolites that, if extracted, might permit the prolongation of life [5]. This put him on a relentless pursuit to create the first artificial kidney.

Early experiments

By 1939, Kolff along with his colleague Dr. Robert Brinkman had designed several prototypes of the artificial kidney that functioned in the laboratory but were not yet suitable for human use. The prototypes utilized a stainless-steel collecting drum and cellophane as the membrane for removing urea and toxins from the bloodstream [6]. In May 1940, the Netherlands was attacked by Nazi Germany, and at that time Kolff moved to a small hospital in Kampen to wait out the war. During this time, by using his knowledge gained from artificial kidney research, he started the first blood bank in Europe, which is still in operation. While in Kampen, Kolff began to find success working on the artificial kidney. He went back and studied the initial attempts to create an artificial kidney by Abel and colleagues in the USA and Georg Haas in Germany. Kolff started his first experiments on blood washing through the wall of inexpensive cellophane “sausage casing” produced from hot dogs in Chicago and heparin as the anticoagulant. This prototype artificial kidney comprised an aluminum frame made of parts from a downed German plane. The materials used were from a local factory in Nazi-occupied Holland [7].

The artificial kidney

**Figure 2:**
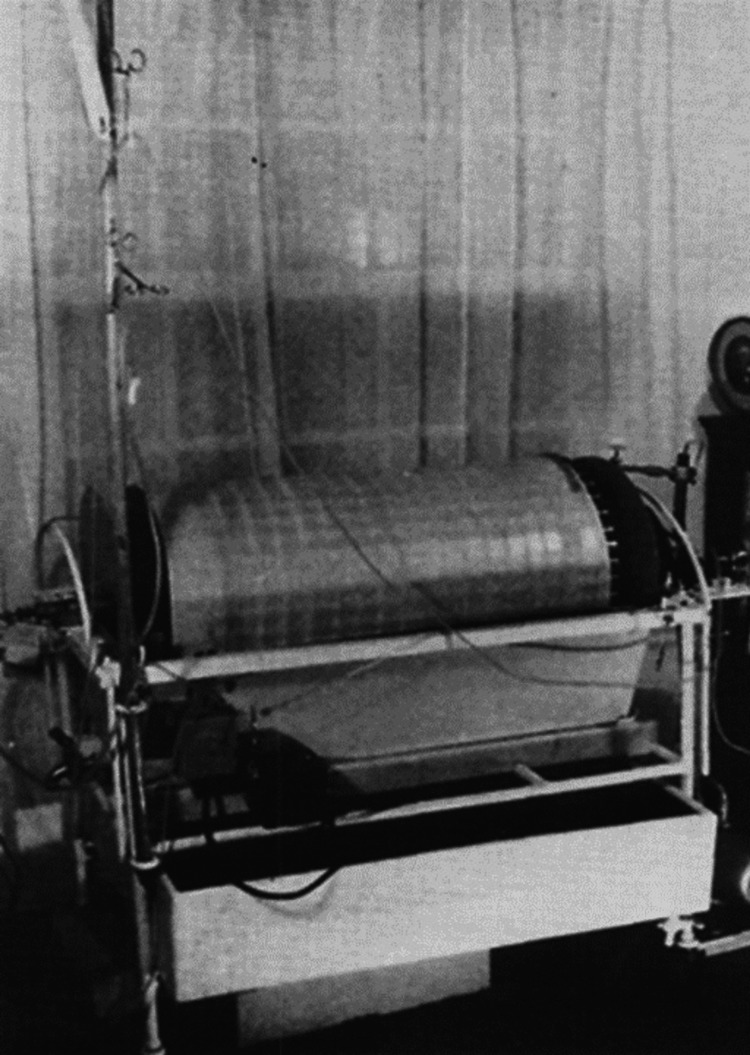
Dr. Kolff’s first artificial kidney (the rotating drum kidney) Source: [4]. Permission to use the figure was granted by Karger Publishers and S.K. Fellner on August 22, 2024 (Kargers Publishers License #5854320942612)

In 1943, with the help of an engineer, Hendrik Berk, Kolff created the “rotating drum kidney (RDK)”, which is regarded as the first working artificial kidney. Kolff’s first device consisted of a rectangular tube with 70-100 liters of salt solution circulated by a Ford Model T water pump in which a sewing machine motor rotated a drum upon which 40 meters of blood-filled cellphone tubing with a surface area of about 2.4 square meters was wrapped with blood moving from top to bottom by gravity (Figure 2) [8]. His paper titled “The Artificial Kidney: a Dialyzer With a Great Area” [9] was published in early 1944 in Acta Medica Scandinavica. During World War II, Kolff’s team treated 16 patients, some with acute and others with chronic kidney failure, with early versions of his artificial kidney. Unfortunately, 15 of 16 patients died, and the effect of the dialysis on the sole survivor was inconclusive [10].

Later years

Despite not achieving success with the first 16 patients, Dr. Kolff continued to make modifications to the device. He needed to suspend dialysis treatments for 14 months until the end of the war. On September 11, 1945, he performed an 11.5-hour dialysis on 80 L of blood in a 68-year-old woman in a coma attributed to hepatorenal syndrome. Dr. Kolff received criticism for treating a Nazi collaborator but stated that as a medical provider, he was not the one who was to decide who to treat based on their background. After the dialysis treatment, the patient awoke from the coma and went on to live for another eight years. She became the first long-term survivor of dialysis. Kolff’s machine is now considered the first modern drum dialyzer, and it remained the standard for the next decade. After the war ended, he donated the five artificial kidneys he constructed to hospitals around the world, including Mount Sinai Hospital in New York.

By 1950, Kolff’s artificial kidney gained worldwide acceptance and the use of dialysis during the Korean War further enhanced its popularity. By the 1960s, Kolff’s invention of the artificial kidney had solved the problem of acute kidney failure; however, it was not seen as the solution for patients with chronic kidney failure. This was addressed by Dr. Belding Scribner at the University of Washington who introduced the concept of connecting the patient to the dialysis machine using a plastic tube inserted into an artery and a vein. After the treatment, the circulatory access would be kept open by connecting to a small U-shaped device made from Teflon. In 1962, Dr. Scribner started the world’s first outpatient dialysis facility. After immigrating to the United States in 1950, Dr. Kolff mentored Dr. Robert Jarvik and together they created the Jarvik-7 artificial heart. Over the years, he mentored many other pioneers in the field of artificial organs and continued to work on artificial organs until he retired in 1997 at the age of 86 years.

## Conclusions

Dr. Willem Kolff was a brilliant physician as well as an inventor and pioneer of artificial organs. He is undoubtedly one of the greatest physician inventors of the last few hundred years. His understanding of physiological and clinical mechanisms combined with his engineering skills made him a hero in the field of artificial organs. Dr. Kolff was listed as one of the 100 most important figures of the 20th century by Life Magazine in 1990. Furthermore, he was knighted in 1970 by Queen Juliana of the Netherlands. Dr. Kolff's work and vision have blazed a trail for new innovative thinking and creativity and continue to have a profound impact on the quality of human life. Thanks to the efforts of Dr. Kolff, millions of people with kidney disease are now able to lead full and productive lives.
